# Progress of the Surgery of the Vermiform Appendix

**Published:** 1901-08-17

**Authors:** 


### Progress of the Surgery of the Vermiform Appendix.

**Appendicitis.**—Southam[1] does not seem opposed to irrigation in cases of suppurative appendicitis and thinks if it is done gently there is little fear of septic matter being diffused throughout the general peritoneal cavity. Renton[2] makes the following divisions of appendicitis:—1. Catarrhal, with no distinct lump or swelling around the appendix. 2. Appendicitis with a swelling which may entirely disappear, either by absorption or the formation of a local abscess, which may require an operation or may burst into one of the viscera, such as the bowel or bladder, or more seriously into the free abdominal cavity. 3. Acute perforative appendicitis, which leads to general peritonitis and the formation of abscess in the general peritoneal cavity. On the subject of abscess in connection with the vermiform appendix Morison[3] has formulated the following conclusions: (1) The early diagnosis of abscess in connection with the vermiform appendix is of great importance. (2) A dangerous abscess may be present without any of the ordinary symptoms or signs of pus formation. (3) The diagnosis is based upon the history of an acute attack of appendicitis and the presence of a definite tender lump. (4) The position of the appendix and the relations of the abscess may be foretold by a careful study of the tumour. (5) The diagnosis of pelvic cases in women is attended by special difficulties. In men and women bimanual examination may clear up an otherwise doubtful case. (6) Spontaneous relief and possible cure may follow the discharge of pus into adjacent bowel. (7) An abscess, though of appendicular origin, may be remote from the appendix and may be residual. Many of such pus collections are due to the localisation of a general peritoneal infection which has been recovered from. (8) Early operation is the proper treatment, and with few exceptions the vermiform appendix should be removed at the same time that the abscess is drained (pelvic cases form the chief exception to this rule). (9) The abdominal opening should be large enough to permit of perfect inspection of all manipulation, and the abscess should be drained from behind. Murray[4] says that, though many of the lower animals possess a long and pointed cæcum, they seldom suffer from an affection at all akin to appendicitis, the probable explanation being that in them it serves a definite physiological purpose. The greater frequency of appendicitis among civilised nations is no doubt due to their more complex diet. Banks[5] considers that many of the cases that are called mild appendicitis nowadays are really cases of typhlitis and perityphlitis; these are of the mildest nature, and recovery without operation is almost invariable. Appendicitis proper may be considered in three stages: (1) Presuppurative; (2) suppurative; (3) quiescent. The presuppurative stage is one from which recovery might be expected in many cases without operation, the treatment being starvation, with fomentation of the flank. The signs of danger are a slight rise of temperature, rapid pulse, increase in the amount of swelling, and drenching sweats; with such the swelling should be incised at once. As soon as the suppurative stage has been diagnosed the pus must be evacuated, when the appendix was practically destroyed, and such patients have no return of appendicular trouble. In the quiescent stage a valuable advance has been made by the removal of the appendix in cases of recurring appendicitis. He would let the patient have three attacks before removing the appendix, because the second attack is often very mild, and only a recrudescence of the original one, which has not quite cleared away. Kümmel[6] states that he has operated upon 740 cases of appendicitis with a mortality of 1 per cent. Richardson[7] narrates three cases of acute diffuse septic peritonitis resulting from appendicitis in which recovery ensued after operation.

**Pseudo-appendicitis.**—Condamin and Voron,[8] referring in the first place to the so-called para-appendicitis in which well-marked symptoms of ordinary appendicitis are due to affections, in most instances of inflammatory nature, of adjacent organs, the appendix itself remaining quite normal, proceed to state that there is a further group of cases in which, though more or less similar symptoms are presented, neither the adjacent organs nor the appendix itself show any signs of disease. To cases of the latter kind the authors give the name of "pseudo-appendicitis." They are usually of a neuropathic character, and occur in hysterical subjects, or may be associated with secondary syphilis, presenting a form of intestinal neuralgia known as "syphilitic typhosis." The local objective signs, such as swelling and fulness, may be present in the hysterical cases, but are absent in the syphilitic variety of pseudo-appendicitis. In both forms there is, as a rule, an absence of depression and of general indications of grave abdominal disease, although fever may be high and the local pain intense. Whenever a case of supposed appendicitis is met with in which, notwithstanding elevation of temperature and severe and well-marked local symptoms, there is an absolute freedom from general reaction, suspicion should be aroused and inquiries made as to the indications of any hysterical or syphilitic influence.

[1] Med. Chron, Nov. 1900. p. 83. [2] Brit. Med. Jour., May 25, 1901, p. 1?53. [3] Lancet, Feb. 23, 1901, p 539. [4] Brit. Med. Jour., March 9, 1901, p. 577. [5] Ibidem, and Lancet, Feb. 23, 1901. p. 554. [6] Brit. Med. Jour., May 4, 1901, p. 1096 [7] Lancet, March 23, 1901, p. 851. [8] Brit. Med. Jour., Jan. 5, 1901, Epitome No. 7.

